# Rosai-Dorfman disease presenting as autoimmune hemolytic anemia in a male child in Palestine: a case report

**DOI:** 10.1093/omcr/omad078

**Published:** 2023-07-18

**Authors:** Yumna Njoum, Lila H Abu-Hilal, Farah Jabbarin, Sami Bannoura, Sameer Mtour, Muaath Itmaizeh

**Affiliations:** Faculty of Medicine, Al-Quds University, Jerusalem, Palestine; Faculty of Medicine, Al-Quds University, Jerusalem, Palestine; Department of Cardiology, Al-Makassed Hospital, Jerusalem, Palestine; Department of Pathology, Al-Makassed Hospital, Jerusalem, Palestine; Department of Cardiology, Al-Makassed Hospital, Jerusalem, Palestine; Department of Rheumatology, Al-Makassed Hospital, Jerusalem, Palestine

## Abstract

Rosai-Dorfman disease (RDD) is a rare, benign non-Langerhans cell histiocytosis predominantly affects lymph nodes and skin. Despite its benign nature, RDD can cause serious hematological complications. A 14-year-old male, presented with 3-month history of hemolytic anemia, lymphadenopathy, hepatosplenomegaly and rash. After thorough investigation, RDD was diagnosed by mediastinal lymph node biopsy that revealed presence of S100 and CD68-positive cells with absence of CD1a confirming the diagnosis of RDD. Treatment involved combination of steroids and Rituximab, which proved to be highly effective. The patient had dramatic improvement and entered remission, with follow-up period of 2 years. It is important to note that although RDD is a rare disease, it causes severe complications, as evidenced by the patient's parameters. Thus, prompt diagnosis and treatment are paramount. Histological diagnosis is of great value, as it helps confirming and guiding treatment decisions. With the right treatment, patients can experience great recovery and quality of life.

## INTRODUCTION

Rosai-Dorfman disease (RDD) has been classified as a rare nonmalignant condition marked by significant histiocyte proliferation in lymph nodes of the head and neck since its initial classification as a distinct pathological entity in 1969 as an uncommon instance of non-Langerhans cell histiocytosis, also known as sinus histiocytosis with extensive lymphadenopathy. RDD has a 1 in 200 000 incidence, affects more men and patients of African descent than other demographics and skin involvement is more frequent in Asian women [1].

Although the primary cause of RDD is still unknown, viral diseases such as the herpes virus, cytomegalovirus, Epstein–Barr virus and Human Immunodeficiency Virus may play a role in its development. RDD is characterized histologically by CD1a negativity, CD68 and S100 positivity. Depending on the afflicted areas, RDD may be categorized into three subtypes: nodal, extra-nodal and mixed [1].

Herein, we present a case of a 14-year-old male who presented with rash that was later diagnosed with RDD complicated by hemolytic anemia.

## CASE PRESENTATION

A 14-year-old male patient presented with a 3-month history of non-painful patches of skin hyper and hypo-pigmentations, distributed over the trunk area.

The patient initially sought medical advice from a dermatologist who treated him with antifungals as a case of fungal skin infection without improvement. At that time, he had no history of fever, night sweats, lymphadenopathy, arthralgia, hematuria or previous episodes.

Over the next months, he developed fatigue and exertional shortness of breath upon activity and reported occasional night sweats with undocumented weight loss. However, he didn’t seek medical advice until 3 months had elapsed, when he developed yellowish discoloration of his sclera associated with dark urine and no change in stool color.

On examination, patient had scleral icterus and hepatosplenomegaly. Laboratory tests revealed hemolysis with hemoglobin levels of 5 g/dl, mean cell volume of 122.7 fL with normal folate and vitamin B12 levels, elevated reticulocyte count of 17.2%, red blood cells (RBCs) 0.79 × 106/μl, with leukocytosis and eosinophilia (25 × 103/μl and 7% respectively) and normal platelet count.

Anisocytosis was found on peripheral blood smear, with teardrops, microcytic hypochromic cells, schistocytes and nucleated RBCs consistent with acute hemolysis. Coombs test was significantly positive, indicating autoimmune hemolytic anemia, high lactate dehydrogenase 576 U/L and bilirubin 4 mg/dl, Direct Bilirubin 0.21 mg/dl, decreased haptoglobin levels and normal liver enzymes.

Erythrocyte sedimentation rate was elevated to 94 mm by the end of the first hour and the C-reactive protein was 19 mg/dl. Body fluids tested negative for microbiology. The viral panel was negative for Epstein–Barr virus, Herpes simplex virus, cytomegalovirus, human immunodeficiency virus, Brucella and toxoplasma.

Coagulation, autoantibodies and kidney function profiles, urinalysis, complement levels were all normal. Immunoglobulin levels were normal except for elevated IgE: 459 IU/ml. Abdominal ultrasound showed a liver span of 14.5 cm and spleen of 16 cm with otherwise normal findings.

Chest and abdominal computed tomography revealed enlarged lymph nodes in the perihilar regions of the lungs with hepatosplenomegaly.

**Figure 1:**
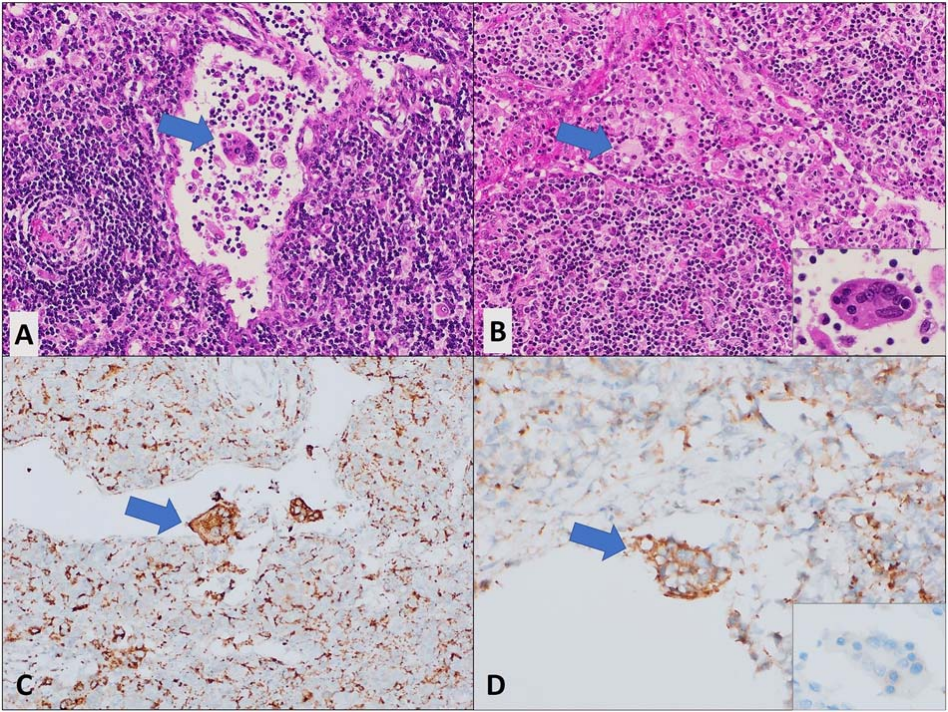
RDD. (A&B) Sections show patent lymph node sinuses containing histiocytes with enlarged nuclei and prominent nucleoli some with engulfed inflammatory cells characteristic of emperipolesis (arrows). Insert shows emperipolesis. (H&E, ×20). Lesional histiocytes are positive CD68 (×20) (C) and S100 while negative for CD1a (insert) (×40) (D.) Immuno-stains supporting the diagnosis.

The symptoms continued with a progressive course. The patient was ill-looking, but stable vitally except for tachycardia. He was maintained on supportive care and was given packed red blood cells. A lymph node biopsy taken from the mediastinum via video-assisted thoracoscopic surgery showed sinusoids filled with S100 and CD68-positive but CD1a-negative, confirming the diagnosis of RDD (Fig. 1).

Methylprednisolone 120 mg was given once daily for 4 days, leading to dramatic improvement in his overall status and substantial improvement in hemolytic parameters within 4 days. He was discharged on Prednisolone 40 mg daily with vitamin D, Trimethoprim/Sulfamethoxazole prophylaxis and rituximab. Follow-up for 2 years showed no disease relapse, and no medications required.

## DISCUSSION

RDD is identified by infiltrating, pale, eosinophilic histiocytes that are S100-positive, CD68-positive and CD1a-negative. Although Rosai and Dorfman initially described this disorder in young Africans, it has been reported globally in individuals of all races. The etiology remains uncertain; nevertheless, the coexistence of autoimmune antibodies during disease activity, coupled with numerous reports providing serological evidence of recent bacterial or viral infections, particularly herpes viruses, suggests a potential association with immune dysregulation secondary to infection [2, 3].

Painless, non-tender cervical lymphadenopathy is the most common presentation, with an average age of onset in the second decade. Fever, night sweats, malaise, weight loss, leukocytosis, elevated erythrocyte sedimentation rate and hypergammaglobulinemia are some of the clinical and laboratory abnormalities that may be present.

RDD can be confused with other histiocytosis, both benign and malignant. However, the unique features of RDD, such as the presence of emperipolesis, the immunocytochemical profile and absence of cellular atypia, along with clinical and laboratory findings, can help distinguish it from other potential diagnoses [4].

Extra-nodal sites are involved along with lymph nodes in 43% of RDD cases. In 23% of cases isolated extra-nodal RDD occurs, with peripheral lymphadenopathy being the most frequent manifestation.

Extra-nodal RDD affects the skin, soft tissue, nasal cavity, paranasal sinuses and skeletal system. The severity of joint disease varied from mild polyarthralgia to disabling arthritis. RDD has been linked to autoimmune phenomena, with hematologic autoantibodies against red cell antigens being most commonly reported [5].

But acute life-threatening autoimmune hemolytic anemia complicating RDD is rare [5, 6].

RDD management varies, and in some cases, like the one with our patient, steroids alone may suffice. However, in refractory cases, chemotherapeutic agents and blood transfusion may be necessary [6].

Multi-organ involvement and immune dysfunction are negative prognostic factors and necessitate treatment. Glucocorticoids and chemotherapy have been used with reported success in some cases where other cases had spontaneous resolution. Methotrexate and 6-mercaptopurine are commonly used [5].

In conclusion, early and appropriate management are crucial to achieving full recovery in RDD patients. Therefore, raising awareness of RDD among healthcare professionals is needed, leading to prompt diagnosis and appropriate management.

## Data Availability

The data supporting this case report's findings are available upon reasonable request from the corresponding author. Due to privacy and ethical considerations, certain data such as patient identifiers or sensitive clinical information may be redacted or anonymized prior to sharing. Requests for data should be addressed to the corresponding author.
